# Incidence of and Risk Factors for Lower Extremity Apophysitis in Children and Adolescents

**DOI:** 10.1007/s40279-025-02328-w

**Published:** 2025-11-03

**Authors:** Niels Wedderkopp, Chinchin Wang, Russell Steele, Jeff Hebert, Christina Rexen, Eva Jespersen, Tina Junge, Tobias Thomsen, Frederik Mølgaard Jensen, Ian Shrier

**Affiliations:** 1 The Pediatric Research Unit, Department of Clinical Research, University of Southern Denmark, Campusvej 55, 5230 Odense M, Denmark; 2 Department of Epidemiology, Biostatistics and Occupational Health, McGill University, Montreal, Canada; 3 Department of Mathematics and Statistics, McGill University, Montreal, Canada; 4 Faculty of Kinesiology, University of New Brunswick, Fredericton, Canada; 5 Department of Sport Science and Clinical Biomechanics, University of Southern Denmark, Odense, Denmark; 6 Department of Rehabilitation, Odense University Hospital, Odense, Denmark; 7 Department of Applied Health Research, University College Lillebaelt, Odense, Denmark; 8 Department of Clinical Research, University of Southern Denmark, Odense, Denmark; 9 Centre for Clinical Epidemiology and Community Studies, Jewish General Hospital, Montreal, Canada

## Abstract

**Introduction:**

Apophysitis is a common musculoskeletal injury in children and adolescents often resulting in pain and deformity such as bony prominences in Osgood–Schlatter, leading to reduced physical function. A concerning consequence of lower extremity (LE) apophysitis is its potential to reduce physical activity.

**Objectives:**

We aimed to describe the incidence of lower extremity apophysitis in children and estimate the association of leisure-time sport (LT-sport) participation and physical education (PE) on apophysitis incidence.

**Methods:**

In a 5.5-year prospective cohort study of primary-school students, parents sent weekly text messages reporting injury occurrences and LT-sport participation data in the participating children. Injury reports triggered subsequent clinical evaluation.

**Results:**

We analyzed data from 878 girls and 792 boys and identified 1265 episodes of apophysitis in 1670 children. Sever’s, Sinding-Larsen–Johansson’s, and Osgood–Schlatter’s diseases constituted more than 99% of LE-apophysitis cases. The median time of injury duration was 3–4 weeks, and the range was between 1 and 45 weeks. The incidence was between 3.2 and 7.0 cases of LE apophysitis per 1000 LT-sport participations. Extra physical education did not increase the risk of apophysitis, whereas children who participated in soccer, handball, basketball, and jump gymnastics were at increased risk (risk ratio [RR] = 2.07–2.74).

**Conclusions:**

Lower extremity apophysitis can result in months of pain. This is important as prolonged periods of injury could reduce LT-sport and exercise participation, thereby decreasing health-related physical activity in childhood. Our results suggest early diagnosis and activity modification in children and adolescents with apophysitis may reduce injury duration, but future intervention studies are required before more definitive causal conclusions can be drawn.

**Trial Registration:**

Danish Data Protection Agency (J. no. 2008-41-2240) and ClinicalTrials.gov (NCT03510494).

**Supplementary Information:**

The online version contains supplementary material available at 10.1007/s40279-025-02328-w.

## Key Points

**Table:** 

Over 5.5 years, we found 1265 cases of lower body growth-related injuries in 1670 children. These injuries were most common in kids who played high-impact sports such as soccer, handball, basketball, and gymnastics.
Playing high-impact sports made it 2–3 times more likely for children—especially boys—to develop lower extremity apophysitis. However, having extra physical education classes at school did not increase the risk.
Our results suggest that it is important to identify these injuries early and adjust activities on the basis of the child’s pain. This might help reduce how long the injury lasts and keep kids active. More research is needed to confirm this.

## Introduction

Musculoskeletal pain is a leading complaint in children and adolescents seeking primary care [1]. Apophysitis is a common musculoskeletal injury caused by repetitive muscle contractions that can injure immature bony and soft tissue structures, often resulting in pain, reduced physical function, and, in some cases of Osgood–Schlatter, visible or palpable bony deformity [2, 3]. A concerning consequence of lower extremity apophysitis is its potential to reduce physical activity below healthy levels [4] during childhood and adolescence secondary to an apophysitis-related increase in body mass index (BMI) [5].

A recent systematic review found the most common sites of apophysitis are the heel (Sever’s disease), distal pole of the patella (Sinding-Larsen–Johansson’s disease), and tibial tubercle (Osgood–Schlatter’s disease) [6]. However, the incidence of apophysitis in the general population is uncertain as most studies to date have focused on athletic or care-seeking populations [6]. Wiegerinck et al. reported an incidence of 3.7 per 1000 children aged 6–17 years in general practice, underscoring the condition’s clinical relevance. However, they did not explore potential risk factors such as sport participation or activity level [7]. This is a potential source of bias, as studies in athletes may miss cases that have ceased sport participation owing to injury, and many people with a musculoskeletal condition do not seek healthcare [8]. Further, several studies have defined injuries as requiring time lost from sport. However, overuse injuries such as apophysitis often have a vague and gradual onset, generating pain but not necessarily causing time lost from sports participation. Consequently, the incidence of apophysitis may be grossly underestimated and has led to suggestions that data collection should include frequent and prospective measures of pain and other symptoms [9].

There is little high-quality evidence elucidating the epidemiology of apophysitis. Repetitive activities, such as running and jumping in sports, such as soccer, basketball, and handball, are assumed to be a common cause of apophysitis [10]. A 2021 systematic review identified sport participation as a potential risk factor for apophysitis but reported that nearly all included studies had serious risk of bias concerns owing to non-prospective designs, unclear potential for reproducibility, lack of confounding control, and problematic data collection methods [6]. Consequently, the review authors called for future studies to examine the role of specific sports and other modes of physical activity on the incident risk of apophysitis.

Therefore, we aimed to describe the incidence of lower extremity apophysitis in children from the general population and estimate the association between leisure-time sport (LT-sport) participation and physical education and apophysitis incidence. Specifically, we described the 5-year incidence and duration of Sever’s disease, Osgood–Schlatter’s disease, and Sinding-Larsen–Johansson’s disease in primary school students. We also estimated the association between LT-sport participation (sport type and frequency) and physical education in school (frequency) and apophysitis incidence.

## Methods

### Study Design and Participants

This was a prospective cohort study nested within the Childhood Health, Activity, and Motor Performance School Study Denmark (CHAMPS-DK), a large pseudo-experimental trial evaluating the effect of increased school physical education on the health of primary school students [11, 12]. The trial methods have been reported in detail elsewhere [11]. Briefly, six primary schools were assigned to deliver six physical education sessions per week, while four control schools delivered the usual curriculum comprising two physical education lessons per week. The extra physical education was designed and planned by teachers and schools with a focus on age-related training, promoting safe, effective, and developmentally appropriate physical development, using the principles of “Team Denmark” (a national organization that supports elite athletes in Denmark). The testing and follow-up of the children at the schools was planned and coordinated with the schools, teachers, and parents. LT-sport participation exposures and apophysitis outcomes were measured weekly during the school year (August to June), except during the 6-week summer and 1-week Christmas holiday periods, from November 2008 to June 2014.

All students enrolled in the participating schools were eligible to participate, except for three children with serious chronic diseases. Ethics approval was obtained from the Regional Scientific Ethical Committee of Southern Denmark (ID S20080047). The original trial protocol was registered with the Danish Data Protection Agency (J. no. 2008-41-2240) and ClinicalTrials.gov (NCT03510494). Parents provided written informed consent, and children provided verbal assent prior to enrollment.

### Leisure-Time Sport Participation

We defined “leisure-time sport participation” as any organized sport activity occurring outside the mandatory school-based physical education curriculum. This included activities such as soccer, handball, gymnastics, or swimming practiced through clubs or local associations.

LT-sport participation data were collected from parents each week via a text messaging system (SMS-Track) [11, 13]. Parents reported the number of times their child participated in leisure-time sport in the previous week. Parents additionally indicated the type of LT-sport (ten options; the nine most popular LT sports in Denmark and one category for “others”) in that specific week. The weekly sampling windows likely limited recall biases inherent to retrospective data [14].

### Apophysitis Outcomes

Each week, parents were sent a text message using SMS-Track inquiring whether their child had experienced any musculoskeletal pain during the past week [11, 13]. When a pain occurrence was reported, parents further indicated the location of pain and whether the report represented a new or continuing problem. New pain occurrences triggered a telephone consultation the next day. If the pain persisted at the time of telephone follow-up, the child was scheduled for a physical examination within the week, which was performed at the child’s school. Initial clinical assessments were conducted by experienced sports clinicians, including physiotherapists and chiropractors. In cases where the diagnosis was uncertain, children were referred to and evaluated at a sports medicine clinic by an orthopedic surgeon with extensive experience in sports-related injuries. He performed a detailed examination and, when necessary, used ultrasound or magnetic resonance imaging (MRI) to confirm the diagnosis.

**Fig. 1:**
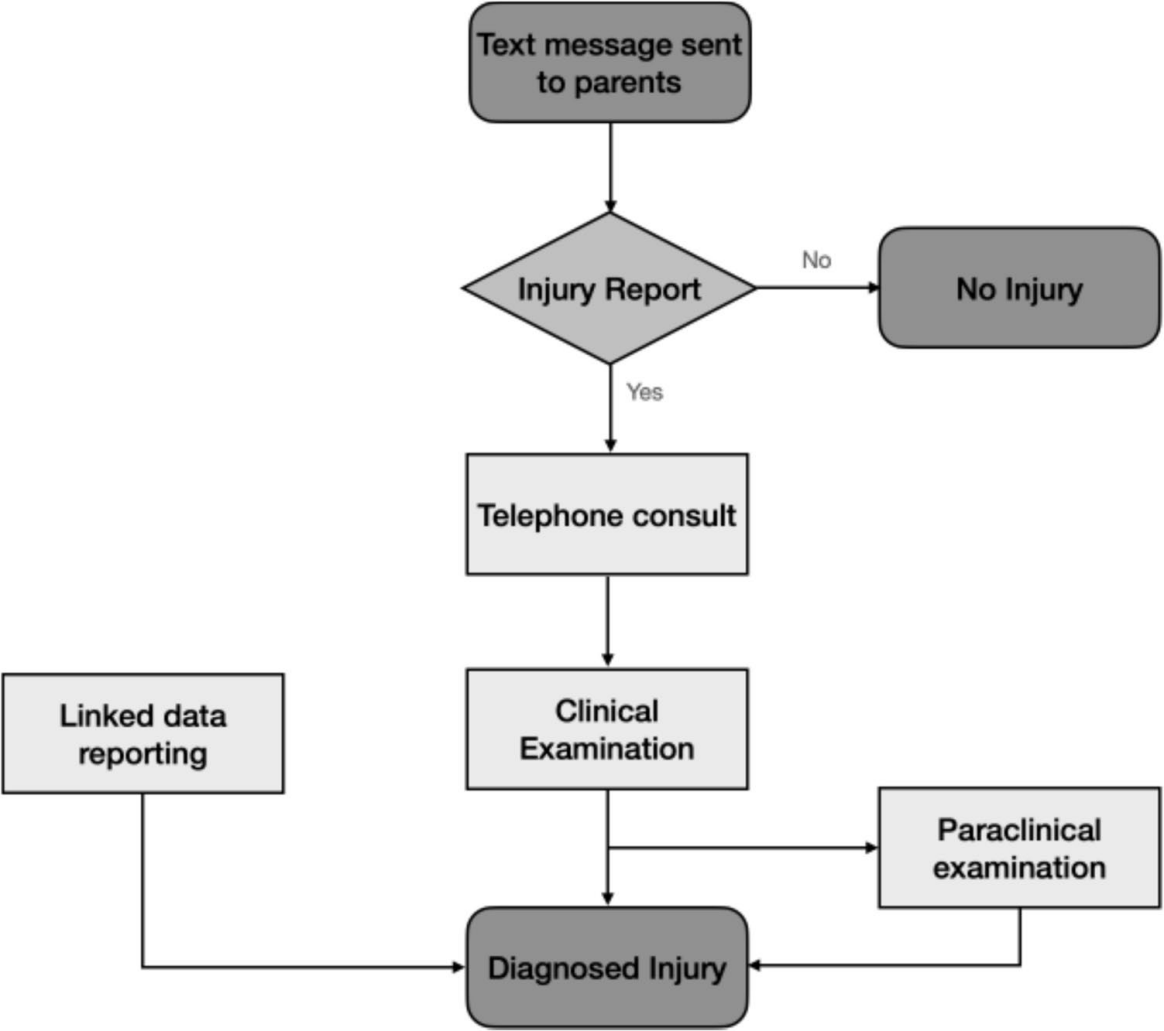
Diagram of the flow of pain and injury registration

Medical evaluation and treatment information from other settings (e.g., emergency departments, general practitioner) were also obtained (Fig. 1). We only considered an injury to have occurred if it was confirmed by a clinician especially trained in diagnosing and treating sport injuries. When the injury was diagnosed, initial treatment consisted solely of information and advice to keep physically active, by avoiding activities that increased tenderness and pain of the injury.

Injury diagnoses were classified according to International Classification of Diseases (ICD-10) criteria. The outcome of interest was lower extremity apophysitis. For our primary analysis, we considered an injury as “healed” when the participant was symptom-free for a week. We evaluated the impact of this decision on incidence rates (a second injury could only occur if the first injury had healed) and injury duration through sensitivity analyses that considered two alternative definitions of healing. Participants had to be asymptomatic and either participated in leisure sport (1) at any frequency again or (2) pre-injury frequency, which we defined as a frequency not less than one participation lower than the average frequency over the 4 weeks prior to the injury.

### Sample Size Calculations

Sample size was calculated prior to the start of the study in 2007. The calculation was carried out taking into account the repeated measurements of the study. Calculation was performed on the planned measurement of physical activity, which we assumed would have the highest standard deviation (SD) and lowest number of repeated measurements. We assumed a change of 50 counts per min over time, and a standard deviation of 200. With three measurements on each subject, 88 subjects in each group (a total of 176 subjects) would be needed to show a difference without taking the cluster effect in the study into account. A cluster factor of 3 was assumed, resulting in 528 subjects needed for the planned analyses. To perform multilevel multivariate analysis, 50 participants would need to be included for the first independent variable and 8 for each of the subsequent variables [15]. With up to 25 independent variables, a “cluster” effect of 3, and a projected drop-out rate of 10%, a minimum of 664 participants were required for the study.

### Data Analysis

The study was an “open natural experiment” with children leaving and entering the study over time. Figure 1 shows the flow of the study with participants leaving and entering the study. The dataset was structured in long (panel) format, where each row corresponds to one child-week observation. Each child could contribute up to 295 weekly observations over the 5.5-year follow-up period (excluding school holidays). To account for repeated measures and temporal variation in exposures and outcomes, we implemented a panel structure in Stata, where ID denotes the child identifier and week the time variable.

As this was an open cohort study, children could enter the study at any time during the enrollment period and could leave the study for various reasons, including withdrawal, school transfer, or end of follow-up. As a result, the dataset contained both left truncation (for participants who joined after the study start) and right censoring (for those who exited before the study ended). This longitudinal, time-varying structure allowed for flexible modeling of exposure-outcome dynamics and was particularly well-suited for the multilevel multinomial logistic regression models with competing risks that we employed. Further, only reported sport participation sessions were included in the analysis; we did not assume that children participated in sport every week. Weeks without reported data were treated as missing. These missing observations were assumed to be missing at random (MAR) and handled using maximum likelihood estimation within multilevel multinomial logistic regression models. This approach allows partial data to contribute to parameter estimation without requiring imputation of outcomes. We believe the MAR assumption is plausible owing to the prospective data structure and the very high response rate of 96.3%.

To describe the distributions of sample demographic characteristics and apophysitis incidence, we reported means with standard deviations (SD) for normally distributed continuous variables and medians with interquartile ranges (IQR) when distributions were found non-normal. Categorical data were reported using numbers and proportions.

We calculated overall incidence rates as the number of apophysitis cases per 1000 person-years for the entire cohort, and the incidence of apophysitis per 1000 sessions of LT-sport participation for those who participated in LT sport. Further, we constructed multilevel multinomial logistic regression models that consider multistate competing risks [16] to identify the effect on apophysitis incidence due to (i) LT-sport participation frequency in general and attributable to specific LT sports played during the last 4 weeks including multi-sport participation as a distinct category and (ii) supplementary physical education. To be sure to identify which LT sport might be associated with the injuries, we excluded weeks where more than one LT sport was performed. Further, for the analyses of the risk of extra physical education, we assumed that children participated in all physical education lessons.

Individuals and school classes were included as random effects to account for clustering and repeated measurements. Multistate models cannot account for participants in two states simultaneously. Therefore, when children simultaneously experienced more than one type of apophysitis or injury, we considered the first injury to represent the outcome of interest. In this analysis, we limited the competing states to occurrences of (1) Sever’s disease, Osgood–Schlatter’s disease, or Sinding-Larsen–Johansson’s disease, (2) any other injury (whether traumatic or overuse injuries), or (3) no injuries. We assessed the risk of entering the different states depending on the specific LT-sport types and the total amount of participation in LT sport in that week and the previous 4 weeks. The variables included in the multivariable analysis were age, sex, LT-sport type, the number of LT-sport participations during the previous 5 weeks, and physical education program (4.5 or 1.5 h per week). We combined the different types of apophysitis, as the model would not converge when running it with the different types of apophysitis owing to too few cases in some LT sports. We tested for interactions between sex and LT-sport participation and calculated the probability of apophysitis in any given week by sex. All analyses were performed using Stata MP 18.5.

### Sensitivity Analyses

As apophysitis is an overuse injury that develops over time and could take time to heal, we performed a sensitivity analysis to account for multistate competing risks by categorizing the participant as returning to LT sport when asymptomatic and participating in leisure sport at either (1) any frequency or (2) at a frequency not less than one participation lower than the average frequency over the 4 weeks prior to the injury. We expected these analyses to reduce the effect of inappropriately assigning two injuries to a participant who was diagnosed with apophysitis and became asymptomatic after greatly reducing workload, only to become symptomatic when activity increased again. If this occurred, our primary analysis would have an artificially (1) high incidence rate and (2) short injury duration. In addition, we estimated the impact of unmeasured confounding by calculating E-values for all significant model results. E-values estimate the minimum magnitude of an unmeasured confounder’s association on the risk-ratio scale with both the exposure and the outcome (assuming equal effect on exposure and outcome), which could explain away the exposure-outcome effect, conditional on the measured covariates [17].

## Equity, Diversity, and Inclusion (EDI)

Our study was a “natural experiment” in Danish schools, including all genders, race/ethnicities, and socioeconomic levels in a Danish municipality. Our author team consisted of four women and six men from different disciplines (medicine, physiotherapy, epidemiology, and statistics), including two authors considered junior scholars. Data collection was similar in all children participating, and we did not alter methods on the basis of regional, educational, or socioeconomic differences of the community in which the study was performed.

## Results

**Fig. 2:**
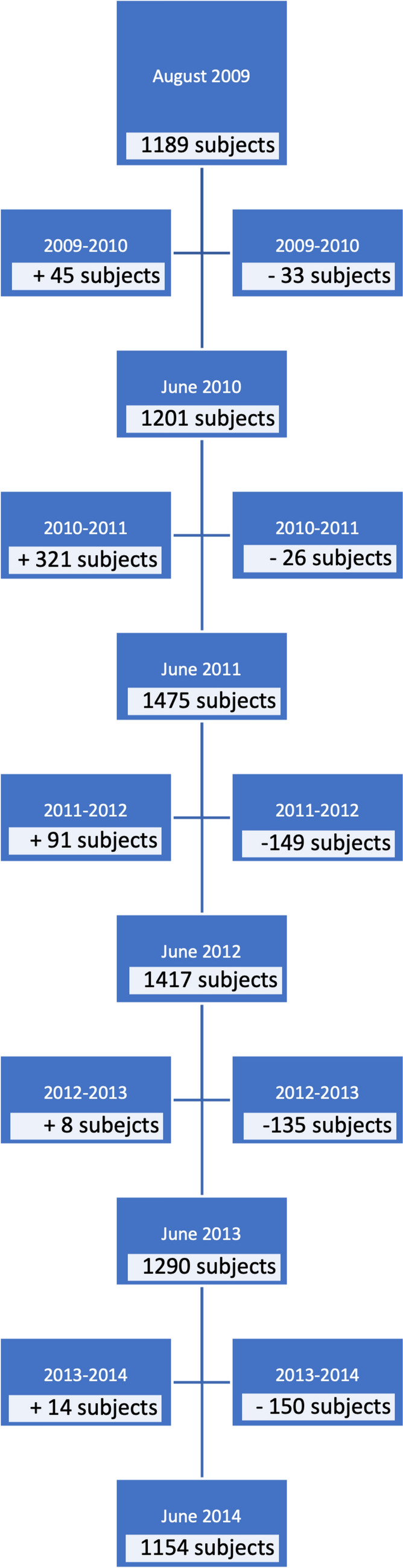
Diagram of the flow of children entering and exiting the study over time

In total, 878 girls and 792 boys (1670 total) participated in the study. Figure 2 shows the flow in and out of the study from August 2009 to June 2014 by school year. Initially, 1189 participants entered the program stepwise from November 2008 to the start of the school year in August 2009. Baseline demographic information is presented in Table 1. The median weeks of participation were 223.5 for boys (interquartile range [IQR]: 134.5–278) and 232 for girls (IQR: 134–278). During the course of the study, we had a 96.5% response rate to text messages (271,298 replies from 281,239 text messages sent).

**Table 1:** Mean (SD) of demographic variables at baseline

	Females (*n* = 878)	Males (*n* = 792)	Any leisure-time sport participation (*n* = 1652)	Never leisure-time sport participation (*n* = 18)	Total sample (*n* = 1670)
Age (years)	9.5 (1.9)	9.6 (1.8)	9.5 (1.8)	9.6 (2.0)	9.5 (1.9)
Height (m)	1.37 (0.12)	1.39 (0.13)	1.37 (0.12)	1.38 (0.13)	1.38 (0.13)
Weight (kg)	32.4 (9.3)	33.2 (9.9)	32.5 (9.2)	33.6 (10.6)	32.8 (9.6)

### Sport Participation Outside of School

Of the 1670 participants, 1652 reported participating in organized LT sports during their leisure time, meaning structured sport activities outside regular school hours. These included club-based or organized recreational sports such as soccer, gymnastics, swimming, or dance. Participation was reported weekly by parents, and children could participate anywhere from 1 to 295 weeks over the study period. Boys participated 2.5 (SD 1.4) times per week and girls 2.4 (SD 1.4) times per week in LT sport. In the majority of weeks with LT-sport participation, 73.5% of the participants participated in one LT sport, 22.9% in two sports, 3.3% in three sports, and 0.3% in more than three sports.

### Apophysitis Incidence

**Fig. 3:**
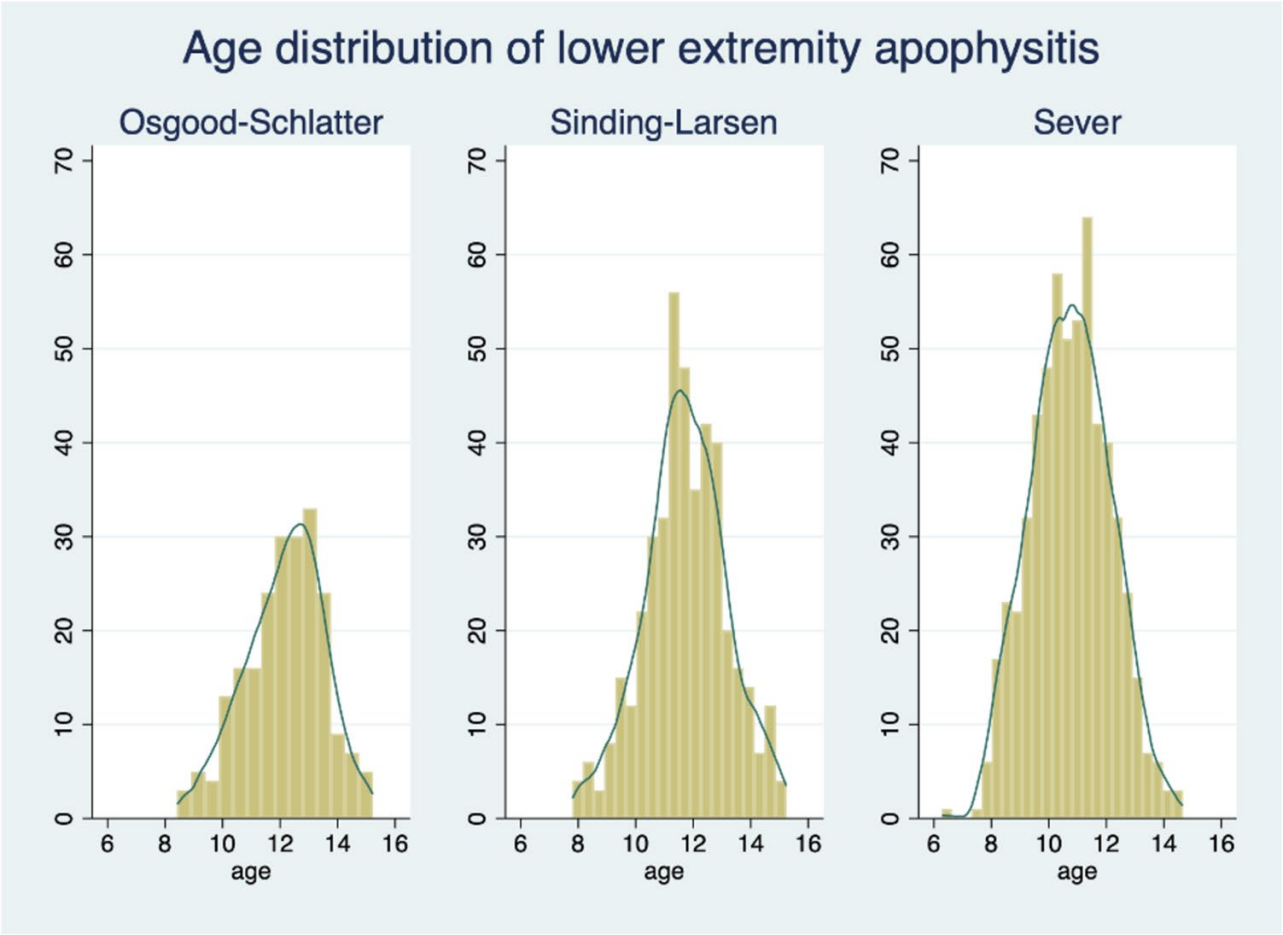
Histograms showing the age distribution at first apophysitis diagnoses, and number of cases by age

The number and proportions of lower extremity apophysitis and other injuries are presented in Table 2. In total, 1265 cases of lower extremity apophysitis lasting between 1 and 45 weeks were diagnosed in 1670 participants. Participants with lower extremity apophysitis constituted 33.2% of all injuries in boys and 25.6% of all injuries in girls. In total, 99.0% of participants with lower extremity apophysitis were diagnosed with Sever’s, Sinding-Larsen–Johansson’s, or Osgood–Schlatter’s disease. The mean age of diagnosis was 10.8 (1.4) years for Sever’s disease, 11.7 (1.4) years for Sinding-Larsen–Johansson’s disease and 12.2 (1.4) years for Osgood–Schlatter’s disease (Fig. 3).

**Table 2:** Distribution of lower extremity apophysitis and other injuries

Injury diagnoses	Males	Females	Total
Sever’s (%)	310 (16.9%)	291 (11.4%)	601 (13.7%)
Sinding-Larsen–Johansson's (%)	182 (9.9%)	247 (9.7%)	429 (9.8%)
Osgood–Schlatter's	113 (6.2%)	109 (4.3%)	222 (5.1%)
Other apophysitis	5 (0.3%)	8 (0.3%)	13 (0.3%)
Other injuries	1228 (66.8%)	1901 (74.4%)	3129 (71.2%)
Total	1838 (100%)	2556 (100%)	4394 (100%)
All values are *n* (%)			

Children with “other apophysitis” were diagnosed with apophysitis of the pelvis (*n* = 2), elbow (*n* = 5), hand (*n* = 2), or foot (navicular) (*n* = 4)

The incidence per 1000 weeks of single LT-sport participation of apophysitis among those who participated in one or more LT-sport sessions is presented in Table 3. The lowest incidence of lower extremity apophysitis was observed in children and adolescents not participating in LT sport (2.4 cases of apophysitis per 1000 weeks or 125.7 cases per 1000 person-years). The highest incidence was found in handball players (7.0 per 1000 weeks, or 364.0 cases per 1000 person-years). Injury duration was non-normally distributed. The median duration for each type of apophysitis ranged from 3 to 4 weeks (Table 4). Participant-weeks where the child participated in more than one LT sport were excluded from sport-specific incidence because we only asked about the total number of sport sessions during the previous week and did not ask how many times they participated in each LT sport.

**Table 3:** Leisure-time sport participation, injury count, and apophysitis cases per 1000 weeks of single-sport participation in the nine most popular sports in the municipality at the time of the study

	Weeks of leisure-time sport participation	Number of lower-extremity apophysitis cases	Apophysitis cases per 1000 weeks of leisure-time sport participation (95% CI)
Other leisure-time sports	42,435	198	4.7 (4.1–5.3)
Soccer	40,322	237	5.9 (5.2–6.7)
Handball	31,781	221	7.0 (6.1–8.0)
Swimming	18,008	57	3.2 (2.4–4.0)
Horseback riding	15,361	52	3.4 (2.6–4.4)
Tumbling gymnastics	12,730	79	6.2 (5.0–7.7)
Dance	8350	29	3.5 (2.4– 5.1)
Rhythmic gymnastics	4959	19	3.8 (2.4–5.9)
Volleyball	3403	14	4.1 (2.4–7.0)
Basketball	3133	20	6.4 (4.1–9.9)
Multiple sports	46,235	256	5.5 (4.8–6.1)

CI, confidence interval

**Table 4:** Injury duration stratified by lower extremity apophysitis diagnosis

	Median (IQR), weeks	Minimum, weeks	Maximum, weeks
Sever’s	3 (2–5)	1	45
Sinding-Larsen–Johansson's	3 (2–5)	1	31
Osgood–Schlatter's	4 (2–6)	1	44

Median, IQR, and range of weeks of injury duration

### Effect of Leisure-Time Sport Participation and Physical Education

Among specific LT sports, participation in basketball, handball, soccer, and tumbling gymnastics had the largest effects (relative risk ratio > 2.00) on apophysitis incidence (Table 5). The effect of additional physical education on lower extremity apophysitis had a nonsignificant risk ratio of 1.07 (95% CI 0.90–1.28).

**Table 5:** Relative risk of lower extremity apophysitis stratified by type of leisure-time sport relative to no leisure-time sport participation with E-values

Leisure-time sport participation status	Relative risk ratio	95% CI	E-value
Other leisure-time sports	1.70	1.36–2.12	2.79
Soccer	2.17	1.75–2.68	3.76
Handball	2.26	1.79–2.85	3.95
Swimming	1.09	0.79–1.51	NR
Horseback riding	1.12	0.79–1.60	NR
Jump gymnastics	2.07	1.54–2.77	3.56
Dance	1.27	0.83–1.95	NR
Rhythmic gymnastics	1.23	0.73–2.07	NR
Volleyball	1.53	0.86–2.75	NR
Basketball	2.74	1.64–4.58	4.92
Multiple sports	1.85	1.50–2.27	3.1
Additional physical education	1.07	0.90–1.28	NR

Values are relative risk ratios and 95% confidence intervals.

As 1 already is included in the 95% CI, no confounding is needed to move the CI to include 1.

NR, not relevant

There was a significant interaction between sex and LT-sport participation, showing that increasing participation in LT sport increased the probability of injuries significantly more in boys than in girls (*p* = 0.021). The probability of acquiring an apophysitis injury in any given week increased with increasing LT-sport participation in boys but not girls. In this study, a girl had an overall probability of 18.5% for developing apophysitis if she participated in leisure-time sports twice per week, 18.7% if she participated four times per week, and 19.4% if she participated eight times per week. In boys, the respective probabilities were 18.2%, 23.3%, and 37.0%.

### Sensitivity Analyses

Similar to our main analysis, there were no clinically meaningful differences in median length of injury, but the maximum length range was increased from 31–45 weeks to 115–144 weeks (see Appendix: Supplementary Table S1). The results from the multilevel multinomial logistic regression models that account for multistate competing risks showed no significant or meaningful differences when we performed our sensitivity analyses.

The E-values ranged from 2.79 to 4.92. This means that substantial unmeasured confounding would need to be present to fully explain away the effects of LT-sport participation on the risk of apophysitis.

## Discussion

We diagnosed 1265 occurrences of lower extremity apophysitis in 1670 participants. The most common type of apophysitis was Sever’s disease, followed by Sinding-Larsen–Johansson´s and Osgood–Schlatter’s disease. Children participating in handball, soccer, tumbling gymnastics, and basketball demonstrated significantly higher odds of lower extremity apophysitis compared with those not participating in sport. While our statistical model adjusts for known confounders and accounts for clustering and therefore suggests a causal relationship, residual confounding due to unmeasured factors cannot be ruled out, and the findings should therefore be interpreted with caution regarding causality.

This is the first study to assess multiple types of lower extremity apophysitis and their incidence, duration, and risk factors relating to LT sport and physical education participation in the general population. The current study advances the existing evidence base, which was previously restricted to populations seeking medical attention [2, 6], and if not restricted to people with Osgood–Schlatter’s disease, could be considered outdated and warrant reevaluation in the case of Sever’s disease [2, 6, 7].

Our results suggest Sever’s disease is the most common form of apophysitis, which differs from a recent systematic review that reported Osgood–Schlatter’s disease as the most common apophysitis in the primary care population [6]. This contrast may reflect the differences in apophysitis incidence between the general and care-seeking populations. For example, if children with Osgood–Schlatter’s disease are more likely to seek care than children with other types of apophysitis, the incidence would be higher in studies conducted in primary care settings or using health administrative data. There are limited epidemiological data available on Sever’s and Sinding-Larsen–Johansson’s diseases, with prior evidence primarily arising from cross-sectional or retrospective studies [6, 7].

We believe our results are likely to represent more accurate estimates of lower extremity apophysitis occurrence in the community than previous studies. However, we encountered two unexpected findings. First, we found that the duration of pain experienced by children with apophysitis was shorter than reported in previous studies. For example, the median duration of pain experienced by the children in this study was between 3 and 4 weeks in our primary and sensitivity analyses. However, the maximum injury duration depending upon injury type (Sever’s, Sinding-Larsen–Johansson, or Osgood–Schlatter) increased from 31 to 45 weeks in the primary analysis to 115–144 weeks in our sensitivity analyses. The results from the sensitivity analyses are consistent with previous studies using care-seeking populations that have reported injury duration up to more than 2 years [10, 18]. Kujala et al. reported that the majority of injuries had a duration longer than 3 months, and up to 70% had to restrict activities for 10 months [10]. Alternatively, Hirano et al. reported shorter duration of 6.3 weeks of missed training, with 66% returning after less than 4 weeks, and 33% missing more than 3 months of training [18], though the study was quite small, with only 40 participants. Our results suggest an even shorter duration than Hirano et al. [18], which may reflect a difference between general and care-seeking populations since our study would likely identify more mild cases that may not require medical treatment. Injury durations in our cohort were shorter than in previously published studies of care-seeking populations [10, 18]. This may reflect the effect of early identification and conservative management (e.g., pain-guided activity modification) as previously described [19]. However, because we did not conduct a specific analysis to quantify this association, this finding needs to be confirmed in future studies. Second, we observed an increasing risk of apophysitis with more frequent LT-sport participation in boys but not in girls. While not a primary aim of the study, this sex-specific difference emerged from our adjusted models. This may reflect biological, behavioral, or sport exposure differences between boys and girls, or have occurred by chance. Because our study was not designed to answer this question (e.g., sample size), we could not stratify our analyses or model interaction effects in depth. Therefore, this observation should be interpreted with caution and warrants confirmation in future studies.

We identified risk patterns using a statistical model that we believe controls for the most likely confounding factors to allow a causal interpretation. In addition, we included sensitivity analyses and E-values to assess the robustness of our associations. These methods support a causal interpretation of the results, but more advanced causal inference methods (e.g., propensity score methods or doubly robust estimators) should be used in future studies.

The consistently elevated risk observed in children participating in high-impact sports such as handball, soccer, basketball, and jump gymnastics likely reflects the biomechanical demands of these activities—specifically repetitive loading through running, jumping, and abrupt directional changes, which place stress on the developing apophyses. Our weekly prospective data allow for more accurate and granular insight than retrospective or care-based studies. In contrast, the additional school-based physical education—despite increasing structured activity—did not show a significant association with apophysitis risk. This program, as described above, was intentionally designed around principles of age-appropriate, developmentally supportive training, which may mitigate mechanical overload. Moreover, prior work has shown that while school-time physical activity increased, total weekly activity levels remained stable, possibly explaining the lack of increased injury incidence [20]. Lastly, the E-values for these sport associations (ranging from 3.56 to 4.92) indicate that substantial unmeasured confounding would be needed to fully explain the observed associations, reinforcing the robustness of our findings.

In addition, our findings, indicating that increased sport participation, particularly in high-impact sports such as soccer, handball, and basketball, is associated with an increased incidence of Sever’s disease, contrast with earlier reports suggesting no clear relationship between physical activity and the development of this condition. For example, Scharfbillig et al. [21] conducted a prospective study and concluded that activity level was not a significant risk factor for Sever’s disease, while Martinelli et al. [22] paradoxically found that lower frequency and intensity of training were associated with higher prevalence in an athletic population. These discrepancies may be due to differences in study design, population characteristics, or definitions of exposure and outcome. In contrast, our study, which uses weekly prospective reporting from a general population sample, aligns with more recent literature suggesting a positive association between physical activity and calcaneal apophysitis. For example, Ceylan and Caypinar [23] reported a high incidence of Sever’s disease in a sport-active pediatric population in Istanbul, while Nieto-Gil et al. [5], in a recent systematic review, identified sport participation as one of the most consistently associated risk factors, which is also in line with our result for participation in multiple sports during the week. These findings are further supported by Arnold et al. [2], who noted repetitive loading during growth periods as a key contributor to overuse physeal injuries such as Sever’s. Taken together, these findings suggest that while the link between sport participation and Sever’s disease may not be evident in all settings, our results reflect a growing body of evidence that supports sport-related mechanical loading as a significant causal risk factor in the etiology of this condition.

### Limitations

We registered weekly LT-sport participation as well as physical education in school, but not other activities, such as playtime and informal activities with potential to cause injury. Our reported injury duration must be interpreted in the context of our study. Injured participants were seen by a clinician within 7–10 days, whereas in general practice, these injuries are usually seen later in the course of injury when the patient seeks healthcare. Whereas general practitioners typically advise patients to stop participating in LT sport for several weeks [10, 24], the clinicians in the current study recommended modified activities that kept the child active but reduced the stress on the apophysis. Our results are applicable when patients are seen very early after onset of symptoms but may not be applicable if seen after weeks of pain. While we observed considerable variation in injury duration, this study was not designed to identify predictors of prolonged cases. Future analyses may explore which sport-related or individual factors contribute to longer-lasting symptoms.

The open cohort structure introduced variation in individual follow-up duration, which could be considered a limitation. However, we were able to address this in our modeling strategy, which incorporated weekly exposure time and used multilevel models to account for repeated measures and clustering.

We were unable to include weeks in which children participated in more than one sport in the sport-specific risk analyses. The large number of possible sport combinations and limited sample size in many of those combinations led to small cell counts and unstable model estimates. This led to inclusion of participation in multiple sports as a separate category in the analysis when estimating sport-specific injury risk, which limited our ability to evaluate the cumulative or interactive effects of specific combinations of sports in multisport participation. While not universal, multisport participation is relatively common in youth and contributes to overall mechanical load—and participating in multiple sports during the week also led to increased injury risk.

### Strengths

This was a relatively large, population-based prospective cohort study of 1670 children followed for more than 5 years. This study was the first to utilize SMS-text follow-ups for ecological momentary assessment to collect LT-sport participation and musculoskeletal pain data in children and adolescents. This method allowed for the recording of all symptomatic apophysitis injuries. During the study period, there was a very high response rate during the weekly SMS follow-ups, with weekly response rates greater than 96%.

All injuries were diagnosed by trained clinicians with knowledge of relevant conditions, which likely helped to minimize misdiagnosis. This is in contrast to the other studies that described population-wise incidences and prevalences. Wiegerinck et al. used general practitioners who were not specially trained in “sport injuries” as our clinicians were [7]. In the systematic review by Haine et al., all studies conducted in the general population, with no mention of treatment provided by clinicians specially trained in sport injuries [6].

Therefore, we believe we achieved our objectives; we have described the incidence of lower extremity apophysitis in children from the general population and estimated the association of leisure-time sport (LT-sport) participation and physical education on apophysitis incidence. Participation in specific high-impact sports—particularly soccer, handball, basketball, and jump gymnastics—was associated with an increased incidence of lower extremity apophysitis, while school-based physical education was not associated with an increased risk. Our results represent the most accurate population-based estimates of lower extremity apophysitis incidence to date. The sensitivity analyses showed that our results are robust to misspecification owing to different definitions of time to healing and unmeasured confounding.

## Conclusions

Our observed association between increased sport participation frequency and higher apophysitis risk aligns with the widely accepted view that repetitive mechanical loading during growth can exceed the adaptive capacity of immature apophyseal structures. High-frequency participation in sports that involve running, jumping, and cutting movements likely amplifies this effect. The findings that both high-frequency and high-impact sport increase the risk of lower extremity apophysitis may be particularly relevant during periods of rapid growth, when bones lengthen faster than muscles and tendons can adapt, leading to increased tension at tendon–bone interfaces. Our study could suggest a potential benefit of early diagnosis and activity modification in reducing injury duration. Therefore, establishing a way to rapidly identify children and adolescents with apophysitis may allow for earlier interventions and improved outcomes. For example, educating trainers, coaches, and parents would hopefully lead to increased knowledge and awareness of apophysitis, earlier diagnosis, a shorter duration of injury, and a faster return to LT sport and physical activity. Future research should investigate strategies to prevent and shorten the duration of apophysitis in children and explore its sex-specific effects on health-related physical activity.

## Supplementary Information

Below is the link to the electronic supplementary material.

Supplementary file1 (DOCX 16 KB)

## Declarations

### Data Availability Statement

Data are available from the CHAMPS Study Steering Committee upon reasonable request. Legal and ethical restrictions apply. Interested parties may contact Professor Niels Wedderkopp (nwedderkopp@health.sdu.dk), and the following information will be required at the time of application: a description of how the data will be used, securely managed, and permanently deleted. In addition a Data Processing Agreement in accordance with “The General Data Protection Regulation (Regulation (EU) 2016/679)”.
