# Supplementary material for: Incidence of and Risk Factors for Lower Extremity Apophysitis in Children and Adolescents
Source: Sports Med. 2025 Nov 3;56(3):793–803. doi: 10.1007/s40279-025-02328-w (PMC13018006; doi:10.1007/s40279-025-02328-w)
Supplement: Supplementary file 1 — Supplementary file1 (DOCX 16 KB) [file 40279_2025_2328_MOESM1_ESM.docx]

**Table 1** Injury duration in sensitivity analysis one, any return to sport, stratified by lower extremity apophysitis diagnosis

|  | | Median (IQR), weeks | | Minimum, weeks | | Maximum, weeks |  |  |
| --- | --- | --- | --- | --- | --- | --- | --- | --- |
| Injury duration in sensitivity analysis one, return to sport at any level, stratified by lower extremity apophysitis diagnosis | | | | | | | |  |
| Sever’s | 4.5 (2–10) | | 1 | | 115 | | |  |
| Sinding-Larsen–Johansson | 4 (2–9) | | 1 | | 136 | | |  |
| Osgood–Schlatter | 5 (2–12) | | 1 | | 136 | | |  |
| Injury duration in sensitivity analysis two, return to sport at the same level as before the injury, stratified by lower extremity apophysitis diagnosis | | | | | | | |  |
| Sever’s | 5 (2–11) | | 1 | | | 115 | | |
| Sinding-Larsen–Johansson | 4 (2–10) | | 1 | | | 144 | | |
| Osgood–Schlatter | 6 (2–13) | | 1 | | | 136 | | |

Median, IQR, and range of injury duration

Means are reported to enable comparisons with other studies
